# High resolution study of the spatial distributions of abyssal fishes by autonomous underwater vehicle

**DOI:** 10.1038/srep26095

**Published:** 2016-05-16

**Authors:** R. J. Milligan, K. J. Morris, B. J. Bett, J. M. Durden, D. O. B. Jones, K. Robert, H. A. Ruhl, D. M. Bailey

**Affiliations:** 1 Halmos College of Natural Sciences and Oceanography, NOVA Southeastern University, 8000 North Ocean Drive, Dania Beach, Florida, 33004, USA; 2 Institute of Biodiversity, Animal Health and Comparative Medicine, Graham Kerr Building, University of Glasgow, Glasgow, G12 8QQ, UK; 3 National Oceanography Centre Southampton, University of Southampton Waterfront Campus, European Way, Southampton, SO14 3ZH, UK; 4 Ocean and Earth Science, University of Southampton, National Oceanography Centre, University of Southampton Waterfront Campus, European Way, Southampton, SO14 3ZH, UK

## Abstract

On abyssal plains, demersal fish are believed to play an important role in transferring energy across the seafloor and between the pelagic and benthic realms. However, little is known about their spatial distributions, making it difficult to quantify their ecological significance. To address this, we employed an autonomous underwater vehicle to conduct an exceptionally large photographic survey of fish distributions on the Porcupine Abyssal Plain (NE Atlantic, 4850 m water depth) encompassing two spatial scales (1–10 km^2^) on and adjacent to a small abyssal hill (240 m elevation). The spatial distributions of the total fish fauna and that of the two dominant morphotypes (*Coryphaenoides* sp. 1 and *C. profundicolus*) appeared to be random, a result contrary to common expectation but consistent with previous predictions for these fishes. We estimated total fish density on the abyssal plain to be 723 individuals km^−2^ (95% CI: 601–844). This estimate is higher, and likely more precise, than prior estimates from trawl catch and baited camera techniques (152 and 188 individuals km^−2^ respectively). We detected no significant difference in fish density between abyssal hill and plain, nor did we detect any evidence for the existence of fish aggregations at any spatial scale assessed.

The spatial distribution of organisms across a landscape can create observable patterns that may be used to infer valuable information about the underlying processes that influence those organisms^1^,^2^,^3^. These patterns may be caused by environmental heterogeneity (e.g. changes in substratum type, or variation along a physical or chemical gradient) or may be linked to biotic interactions between individuals (e.g. shoaling behaviours in fish). These various processes have been shown to predominantly produce aggregated distributions of organisms in terrestrial, freshwater and marine ecosystems^4^,^5^.

The abyssal realm (3,000–6,000 m depth) covers 85% of the world’s seafloor and forms a vast, globally contiguous habitat^6^. Abyssal demersal fish are typically large, mobile scavengers and predators, and are likely to be significant in the maintenance of ecosystem structure and function. Scavenging demersal abyssal fish, such as the globally-distributed *Coryphaenoides armatus*^7^, may be particularly important in redistributing energy and nutrients across the seafloor^8^,^9^, while predators of benthopelagic prey may enhance energy transfer rates between the benthic and pelagic realms^10^,^11^. Given the vast size of the abyssal realm and the circumglobal distributions of some demersal fish, understanding the ecology of abyssal fish has global relevance. Predatory fish in shallow waters can strongly influence the population structure of their prey^12^, though less is known about the importance of top-down drivers in deep waters. On the NE Pacific abyssal plain, the abundance of invertebrate megafaunal prey was positively correlated to the abundance of abyssal grenadiers following a time-lag^13^, and scavenging species (e.g. *Coryphaenoides armatus*) were positively correlated to temporal variations in carrion availability^14^. The latter two studies imply that bottom-up processes may be more important in abyssal ecosystems than top-down processes, but a lack of data from other regions or species limits these generalisations.

Little is known about the ecological roles of abyssal fish or their spatial distributions. At broad scales (>100 km), correlations have been reported between primary productivity in the surface waters and the abundance of some abyssal fish. Trawl and baited camera studies conducted in the NE Atlantic suggest that abyssal fish abundances may be positively correlated with basin-scale latitudinal gradients of primary productivity^15^,^16^,^17^. Henriques, *et al*.^18^ noted a similar link between variations in regional-scale primary production and density of macrourids on the Cape Verde Terrace and Abyssal Plain, driven by upwelling off the coast of Senegal. The fine-scale (~1 km) distributions of abyssal fish are virtually unknown, though some inferences can be made from existing studies. The use of baited cameras has demonstrated that some scavenging taxa will form temporary, dense aggregations at bait^19^, which are similar to those observed at whale carcasses^20^. However, essentially no data currently exist describing how abyssal fish are distributed when carrion is not present. Priede, *et al*.^21^ estimated that only c. 22% of fish species on the Porcupine Abyssal Plain (PAP) were scavengers, meaning that no fine-scale spatial data exist for the remaining 78% of abyssal fish species that are not attracted to bait. Determining how abyssal fish are distributed would provide valuable data to better understand their role in the ecosystem, and infer which ecological processes may be significant at particular spatial scales.

It is increasingly apparent that abyssal ‘plains’ are highly heterogeneous landscapes across a range of spatial scales^22^,^23^. Of particular relevance to fish populations may be the numerous abyssal hills (topographic features >100 m high). Abyssal hills are estimated to be the dominant landform on Earth^24^, with Wessel *et al*.^25^ speculating that there may be as many as 25 million uncharted abyssal hills (>100 m high) in the global ocean based on the size-frequency distributions of 13,000 known seamounts. Abyssal hills have the potential to generate considerable spatial heterogeneity at scales ranging from tens of metres to several kilometres and to influence sediment habitat characteristics^23^ and the distributions of invertebrate megafauna^22^. Understanding the ecological impact of such features is a significant concern in the effective spatial management of deep-water resources.

Abyssal hills may locally increase secondary productivity by modifying the oceanographic conditions surrounding the hills, as established for larger seamount features^25^,^26^. Seamounts can generate complex local current regimes, which in turn can lead to increases in both primary and secondary productivity. It is possible that even relatively small hills may enhance local food resources and/or secondary productivity, and so influence the distribution of abyssal fish. Durden *et al*.^22^ found that abyssal hills ranging in elevation from c. 100–500 m supported over three times the biomass of invertebrate megafauna than was observed on the abyssal plain at the PAP. If the abundance of abyssal fish does correlate generally with invertebrate megafaunal abundance^13^, then we might expect even relatively small abyssal hills to support a higher abundance of fish than the plain.

The present study was designed to investigate the composition and spatial distribution patterns of the abyssal fish assemblage on the PAP, both in the vicinity of a small (c. 240 m high) abyssal hill, and over the level seafloor of the PAP benthic long-term (30-year) study site^27^. These objectives have only recently become achievable through the development of autonomous robotic vehicles capable of full ocean depth operations^28^ Specifically, we employed the Autosub6000 Autonomous Underwater Vehicle (AUV) to conduct a spatially explicit, ultra-large-scale photographic transect survey (extending over c. 160 km^29^). Here, we use the resultant data to establish: (a) the best estimate of ‘true’ abyssal demersal fish density available to date; (b) the influence of survey method on apparent fish species composition and density; (c) the impact of abyssal hill topography on fish populations; and (d) the first assessment of natural spatial dispersion pattern in abyssal demersal fish

## Results

### Densities and Assemblage Composition

**Figure 1:**
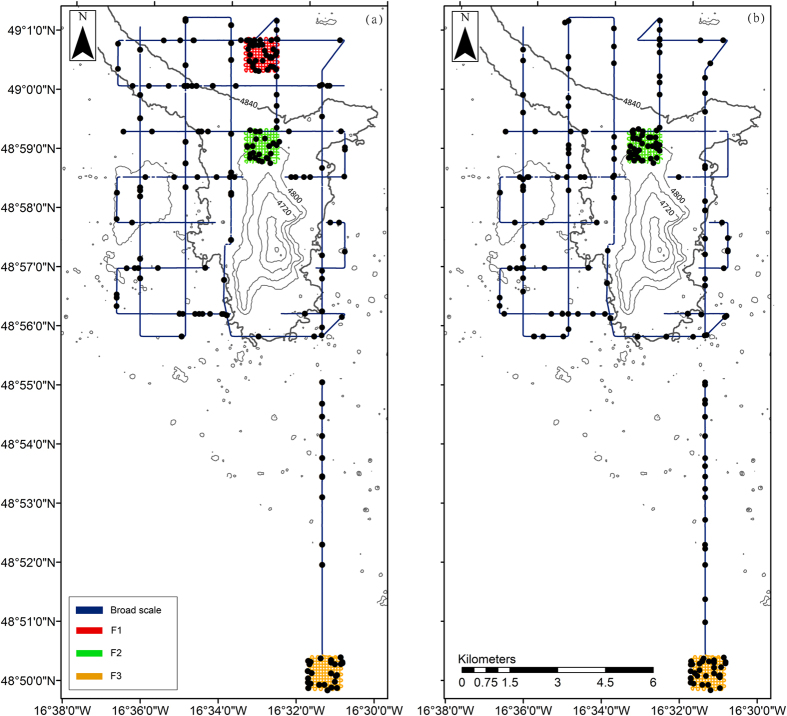
Locations of all fish (black circles) observed by: (**a**) the oblique camera and (**b**) the vertical camera during each AUV survey. Depth contours are marked at 40 m intervals. Projection: UTM Zone 28. Created with ArcGIS v. 10.1 (http://www.arcgis.com).

The AUV surveys recorded 203 individual fish from the oblique camera surveys (0.29% of the images) and 194 from the vertical camera surveys (0.11% of the images) from a total of 11 taxa (Supplementary Tables S1 and 2). The fish fauna was dominated by the macrourids *Coryphaenoides profundicolus* and *Coryphaenoides* sp. 1 (which was likely *C. leptolepis* and *C. mediterraneus*; Supplementary Table S3) that comprised 41.1% and 37.2% of the total fish density based on the oblique images and 42.4% and 40.5% based on the vertical images*. C. armatus* and *Histiobranchus bathybius* were also common, comprising 7.5% and 4.6% of the total fish density respectively from the oblique images and 7.1% and 2.6% from the vertical images. Six unidentifiable individuals (Indet. sp.) were recorded. The locations of all fish observed during the surveys are shown in Fig. 1. The bootstrapped mean density of fish was estimated to be 369.3 (bootstrap 95% C.I. = 315.3, 423.7) individuals km^−2^ from the oblique images and 717.1 (bootstrap 95% C.I. = 614.0, 818.8) individuals km^−2^ from the vertical images. The SHRIMP survey recorded 11 fish from c. 4 hours of oblique-view video footage.

Multivariate analysis of the untransformed data showed a significant difference between the fish communities detected by each AUV camera type (one-way ANOSIM: R = 0.865, p = 0.029), where the vertical camera typically observed higher densities of fish (Table 1). SIMPER analysis conducted on the untransformed data showed that the vertical camera recorded higher densities of most taxa, with the exception of *Coryphaenoides* spp. and *Histiobranchus bathybius*. No significant difference was found when the analysis was conducted on presence-absence data (one-way ANOSIM: R = 0.453, p = 0.057).

### Distribution Patterns

**Figure 2:**
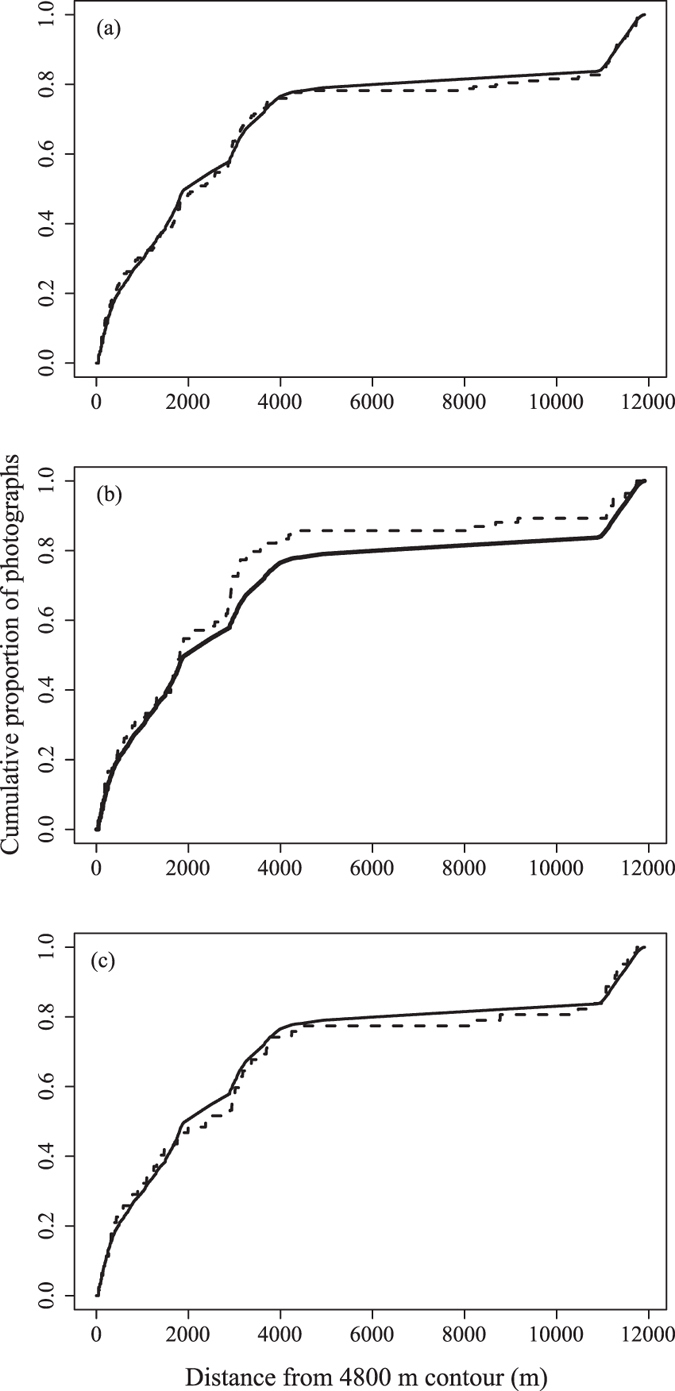
Example cumulative frequency plots showing the distances of every oblique image to the 4,800 m hill contour according to whether they contained fish (dashed line) or not (solid line). (**a**) Total fish fauna; (**b**) *Coryphaenoides profundicolus;* (**c**) *Coryphaenoides* sp. 1. No significant differences were found between the distributions of images containing fish and those that did not (Kolmogorov-Smirnov test: p > 0.05).

The locations of all fish observed during the surveys are shown in Fig. 1. Bootstrapped Kolmogorov-Smirnov analysis detected no significant differences in the distances to any depth contour between images containing fish and those containing no fish (p > 0.05). This was also true when comparing images containing *Coryphaenoides* sp. 1 or *C. profundicolus* to those that did not (p > 0.05), and suggests that the distributions of the fish fauna were no different to random with respect to the location of the abyssal hill. Full statistical outputs are provided in Supplementary Tables S4–S6. Figure 2 compares the cumulative distributions between images that did not contain fish with those that did. The distances of each oblique image to the 4,800 m hill contour are used as an example, but results were similar at all depths.

Bootstrapped densities of the total fish fauna and two dominant macrourids were estimated from the fine-scale surveys using both the oblique and vertical cameras. In most cases the 95% C.I.s overlapped, suggesting no significant differences in density estimates between the plain north of the hill (F1), the hill flank (F2) or the plain at the PAP benthic time series site (F3; Table 1). An exception was the higher density of *Coryphaenoides profunidcolus* observed by the oblique camera to the north of the hill (271.2 individuals km^−2^; 95% C.I. = 150.4, 394.3) compared to the PAP benthic time series site (76.1 individuals km^−2^; 95% C.I. = 20.0, 132.2). No significant differences were detected from the vertical camera surveys. Bootstrapped density estimates calculated from combined broad- and fine-scale survey data showed no significant differences between densities of fish observed on elevated topography (<4840 m) when compared to the abyssal plain (>4840 m) for the total fish fauna or either of the two dominant species (Table 2).

**Figure 3:**
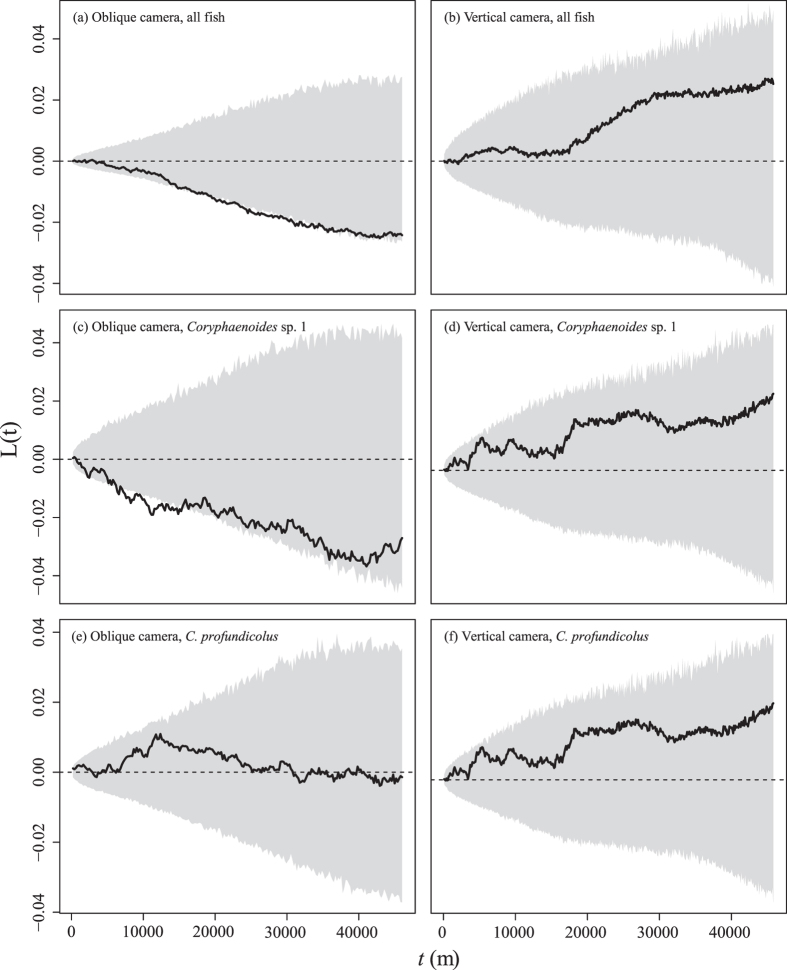
1D Neighbour K plots showing the distribution patterns of all fish (a,b); *Coryphaenoides* sp. 1. (c,d); and *Corphaenoides profundicolus* (e,f) at different spatial scales. *t* (m) is the size of the spatial “window” used to estimate *L(t)* around any given fish in the distribution. The normalised observed values (solid line) are shown against 95% C.I.s (grey region).

No significant clusters of fish (all species), *Coryphaenoides* sp. 1 or *C. profundicolus* were detected in the broad-scale transects. Significant over-dispersion (i.e. uniform spatial dispersion) was detected in the oblique camera data for the total fish fauna and *Coryphaenoides* sp. 1 at scales of approximately 20–35 km and 8–15 km respectively (Fig. 3). No other distributions were different to random. Analysis of the fine-scale survey grids indicated that all of the observed variance to mean ratios fell within the expected 95% C.I.s generated by Monte-Carlo sampling for the total fish counts and for both *Coryphaenoides* sp. 1 and *C. profundicolus* separately. This provided no evidence that the observed numbers of fish per grid cell were different from random for either the oblique or vertical cameras at this scale (c. 90 m^2^). No significant effects of “latitude”, “longitude” or “depth” on the occurrence of fish were detected over any of the fine-scale surveys (GLM: p > 0.05). Full statistical outputs are provided in Supplementary Tables S7–S9.

## Discussion

Observing how organisms are spatially distributed can provide valuable data about how they respond to their physical environment and interact with other individuals^1^,^30^. In the present study, we estimated the total fish density within the study area to be between 423 and 763 individuals km^−2^. Previous trawling studies at the Porcupine Abyssal Plain have reported total fish densities of 152 individuals km^−2^ 31 and similar species composition^21^, though smaller taxa (e.g. *Bathytroctes* spp.) were not observed by the AUV. Priede and Merrett^32^ reported estimated mean densities of 188 (range: 83–741) individuals km^−2^ from baited camera studies at the PAP. Both prior values are of comparable magnitude to, though rather lower than, our present estimate. The high resolution of Autosub6000 surveys compared to trawls or baited cameras, as well as the accuracy with which the locations of individual fish and survey effort can be estimated suggests that AUV surveys could be an excellent tool for future surveys of deep-water fish communities.

Increases in primary or secondary productivity over elevated topography (e.g. seamounts) can lead to aggregations of fish^33^, but no evidence was found in the present study to suggest that fish densities were significantly greater on or close to the abyssal hill (411–795 individuals km^−2^) than on the surrounding abyssal plain (354–723 individuals km^−2^). Durden *et al*.^22^ detected significant and substantial (x3) increases in benthic invertebrate biomass on abyssal hills at PAP, attributing the difference to an additional lateral supply of particulate organic matter (POM) to the hill sites. While we would not expect the fish fauna to respond to the enhanced POM supply, we might expect them to respond to the enhanced potential prey biomass. Conversely, if scavenging on large food falls^9^,^14^ or predation on benthic or pelagic macrofauna^34^ predominates resource use by the observed fish fauna, then a mismatch with the distribution of invertebrate megafaunal biomass would not be surprising.

The distributions of fish were not significantly different from random in any of the fine-scale analyses or in four of the six broad-scale analyses. The oblique camera showed significant over-dispersion of the total fish fauna and of *Coryphanoides* sp. 1 at spatial scales ranging from c. 8–35 km in the broad-scale surveys. The fact that the over-dispersion was detected at such a large (one-dimensional) scale makes them difficult to interpret in the context of the survey design (a two-dimensional grid), particularly since over-dispersion was not found in the vertical camera data. Overall however, the findings from the present study imply that the presence of the hill did not influence the distributions of the total abyssal fish fauna over the spatial scales measured.

At the PAP, *Coryphaenoides armatus* and *Histiobranchus bathybius* are common scavengers that readily form dense aggregations in the presence of carrion^16^,^35^. However, no carrion was observed in the present study and there was no evidence that these (or any other) species formed similar aggregations in its absence. These findings support previous observations from baited camera and acoustic tracking studies at the PAP which indicated that *C. armatus* and *H. bathybius* were unlikely to form shoals or exhibit any social behaviour beyond forming aggregations at bait^8^,^9^,^32^,^35^,^36^. We anticipate that such knowledge of the spatial distributions of abyssal fish will inform future studies of the ecology of abyssal ecosystems (e.g. how energy and carbon are distributed over the seafloor), and provide insight into the life-histories and behaviour of demersal fish (e.g. foraging behaviours and intra- or interspecific interactions).

Random distributions of organisms are unusual in nature. The vast majority of species examined in terrestrial, freshwater and marine ecosystems show clustered (or aggregated) distributions and randomness is rare^4^,^5^,^37^. In large part, this is because natural landscapes are themselves heterogeneous, and because areas that are close to each other are more likely to be similar than areas that are far apart^38^. Consequently, spatial distribution patterns are strongly influenced by the scale at which observations are made, with different processes often producing patterns that are only evident at a particular scale. In general, environmental heterogeneity is believed to be the dominant process influencing spatial patterns at broad scales and typically leads to clustered faunal distributions. At smaller scales, environmental variability is reduced and observed distribution patterns are most frequently driven by interactions between species or individuals^1^. However, stochastic dynamics can also play an important role, especially at fine scales^39^,^40^, and the definitions of “broad-” and “fine-scale” themselves will also vary with the size of the individuals being considered. The results from the present study suggest that abyssal fish did not form aggregations at spatial scales of <1 m to c. 45 km. This finding may have important consequences for understanding how faunal distribution patterns arise both in the deep sea and in other ecosystems.

Knowledge of a spatial pattern is not sufficient on its own to identify the underlying causal mechanisms, as multiple processes may be able to generate any given pattern^41^. The random distribution of abyssal fish observed in this study has three possible explanations. The first is that the density of abyssal fish is simply too low for patterns to be detected at the spatial scales considered here (<1 m–10 km). This is suggested by the fact that only two taxa (*Coryphaenoides* sp. 1 and *C. profundicolus*) had high enough densities to be analysed individually, and is likely to remain a problem for spatial studies of low-density organisms in the deep sea. As *Coryphaenoides* sp. 1 is almost certainly two species, which cannot be discriminated in photographs (*C. mediterraneus* and *C. leptolepis*), potential differences in their niches could hide spatial patterns in either species. The second explanation is that neither environmental heterogeneity (i.e. proximity to the hill) nor biotic interactions were strong enough processes to alter the distributions of fish over the spatial and temporal scales considered here. Effectively, this hypothesis suggests that the environment at the PAP is spatially homogeneous from the perspective of the fish and that the presence of other individuals has no influence on their distributions. Previous studies have shown that broad-scale aggregations of abyssal fish can occur over 100s of kilometres and are potentially correlated with overlying productivity gradients^15^,^16^,^17^,^18^. It is therefore possible that the spatial scale at which aggregations of abyssal fish occur is far larger than was considered in the present study. The third hypothesis is that spatial processes do operate at the observed scale, but generate patterns that are indistinguishable from random. Such a result may be produced if strong attractive and repellent forces operated simultaneously across the study area for example, each cancelling out the effects of the other. For example, if high-value patches of prey are relatively small and distributed randomly across the study area, they may attract fish predators (promoting aggregations) while competition between fish may impede the formation of aggregations. In this case, the fish would not truly be distributed at random (although the resulting pattern may be indistinguishable from a random one). As one reaches a broad enough scale where differences in sinking carrion or prey abundances might arise (e.g. over oceanic biogeographic provinces^42^), then scavenging fish distributions may relate to such differences. Further empirical and theoretical studies will be required to distinguish between the proposed hypotheses.

Hill topography does appear to influence both the local supply of POM and the accumulation of seabed sediments^22^,^23^. Our results for fish stand in contrast to those observed with invertebrate megafauna at PAP, where greater biomass was observed on hills than the surrounding plain^22^. Large food falls (carrion items) are orders of magnitude greater in mass than POM and sediment particles, consequently, they are likely to sink faster and be less subject to lateral transport in topographically enhanced bottom water currents that are assumed to drive the variations in invertebrate biomass. This may explain a lack of response in scavenging fish (i.e. species attracted to bait). However, additional data are required to determine whether predatory fish distributions may by influenced by macrofaunal or benthopelagic prey distributions for example, or by other environmental variables which may vary temporally, or over broader spatial scales than were measured here.

Despite the large size of our survey, the low density of abyssal fish suggests that the results of the present study must be treated cautiously until further observations can be made, ideally including surveys in the vicinity of other topographic features. For example, it is conceivable that other hills and seamounts in the vicinity of the current study could have exerted additional influence on the observed results^23^. Similarly, the present study was conducted over a short time period (days). While this will have reduced the influence of temporal variability, it does mean that our results may not be representative of the average annual condition. The PAP region is subject to seasonal and interannual change in food supply^43^, and the fauna may distribute themselves differently as food availability changes. Repeating the survey over a longer time period would help to provide clarification of the spatial distributions observed in the present study and determine whether they are temporally stable or not.

In the present study, the total density of fishes was consistently higher in the vertical than the oblique camera estimates (Table 1), which is in opposition to expectations of vehicle avoidance by fish. Behavioural responses to both remotely-operated and manned submersibles are an important source of bias when estimating the densities of mobile fauna and are particularly problematic when they occur outside the field of view^44^,^45^. Behavioural responses can take several forms, but fundamentally include those where a response can be directly observed, or those where a response occurs outside the observed area and cannot be seen. However, it is impossible to determine whether avoidance behaviours occurred outside the field of view, or whether particular taxa may have been attracted to Autosub6000. Previous studies examining the responses of deep-sea fish to survey vehicles have reported numerous factors that appear to induce behavioural responses in different species. For example, strobe lighting has been shown to induce both attraction and avoidance behaviours in different species at depths <1,500 m^46^,^47^,^48^. In the abyssal NE Pacific, towed camera observations showed that *Bathysaurus mollis* (Synodontidae) displayed a strong escape response to approaching equipment, while macrourids did not respond until the camera was very close^13^. Further assessment of species responses to Autosub6000, perhaps using video footage, would be required to understand the level of bias inherent in any results.

The present study successfully employed a deep-ocean autonomous vehicle (Autosub6000) to map the fine-scale distribution of abyssal demersal fishes. The results from this study suggest that abyssal fish did not form aggregations across the PAP, neither at the PAP benthic time series site nor on an abyssal hill. While it is not currently possible to attribute these observations to any underlying causal process, they suggest that abyssal fish do not naturally form dense, shoal-like aggregations in the absence of a strong stimulus (such as a carrion food-fall). These observations provide evidence to support the predictions of previous investigators that abyssal scavengers are not gregarious and do not aggregate over the seafloor^8^,^9^,^32^,^35^,^36^. Similarly, these results provide no evidence to suggest that the region surrounding the abyssal hill considered in the present study supported a greater abundance or different composition of abyssal fish compared to the open abyssal plain at the PAP benthic time series site. However, further study will be required to determine whether these patterns are also observed at other locations or if they may vary over time.

## Methods

### Photographic Surveys

**Figure 4:**
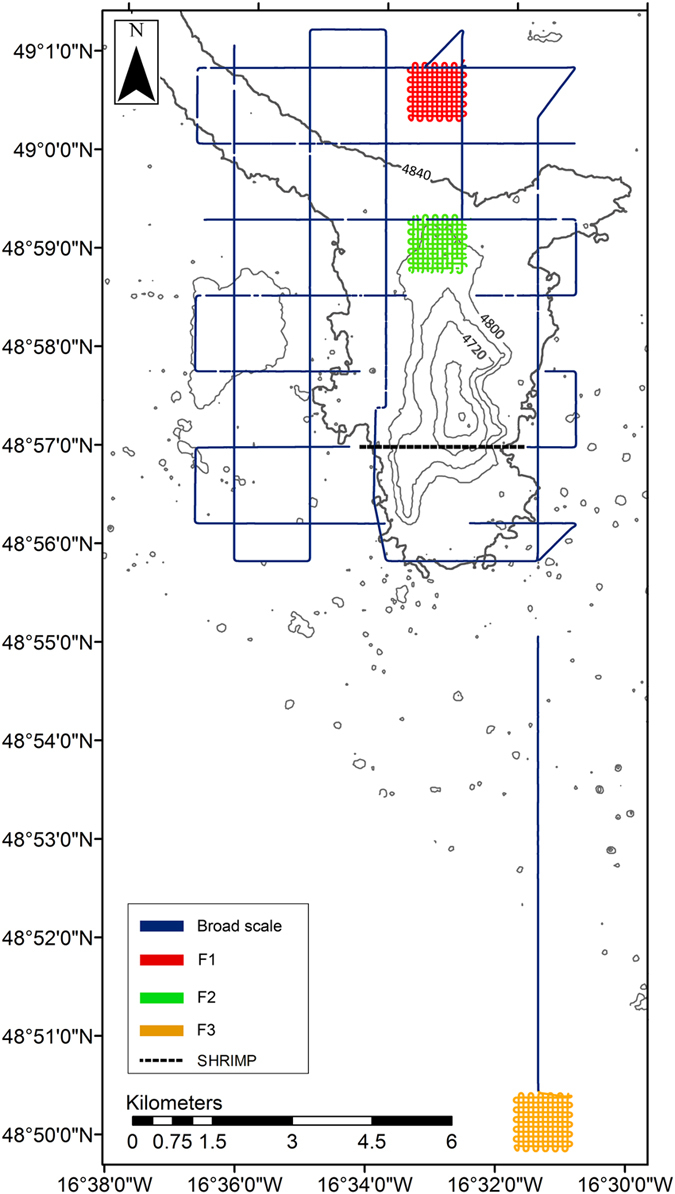
Locations of the Autosub6000 and SHRIMP surveys at the hill site and PAP benthic time series site (F3). Depth contours are marked at 40 m intervals (Projection: UTM Zone 28). Created with ArcGIS v. 10.1 (http://www.arcgis.com).

An autonomous underwater vehicle, Autosub6000^49^ was deployed at the PAP in July 2012 during research cruise RRS *Discovery* 377 to conduct photographic surveys of the benthic fauna in the vicinity of an abyssal hill^29^,^50^. The hill was located c. 15 km to the north of the PAP benthic long-term study site^51^, with a summit at c. 4,615 m water depth (c. 240 m above the abyssal plain). Photographic transects were conducted across two spatial scales (Fig. 4). The broad scale survey comprised a 10 × 10 km survey grid around the hill, with 1 km spacing between tracks. Fine-scale surveys covering 1 × 1 km grids were conducted on the abyssal plain to the north of the abyssal hill (F1) and on the northern flank of the hill (F2) with c. 90 m track spacing. A third fine-scale survey was conducted at the PAP benthic long-term study site (F3) followed by a longer transect (c. 12 km) connecting it to the abyssal hill surveys. A total estimated seabed area of 0.482 km^2^ was surveyed with an oblique-facing camera and some 0.258 km^2^ with a vertically-mounted camera^29^ (Table 3). The cameras were operated simultaneously, such that the vertically viewed area effectively represented a subset of the obliquely viewed area.

Photographs were taken using two identical Point Grey Research Inc. Grasshopper 2 cameras (5MP resolution; 2,048 × 2,448 pixels): a colour camera mounted vertically on the underside of the AUV, and a black and white camera mounted at an oblique angle (35° below horizontal) at the front of the vehicle. Photographs were taken at 0.87 second intervals to produce near-contiguous images of the seafloor. At the target survey altitude of 3.2 m above the seafloor, images from the oblique camera represented a field of view of c. 16.5 m^2^ while the vertical camera represented c. 2.4 m^2^. The position of the AUV was calibrated against the ship’s DGPS position via ultra-short baseline (USBL) tracking at the start of each deployment. Thereafter, the vehicle’s position was determined by inertial and bottom-locked Doppler navigation and recorded at two-second intervals, along with measurements of the vehicle’s pitch, roll, yaw, altitude above the seafloor and heading. These data were subsequently used to calculate the location of each photograph and observed fish, as well as the seabed area surveyed by each camera using a 3D rotation matrix calculation^29^ by estimating the spatial position of the corners of each image using MATLAB^®^ software (Release 2013a (8.1.0.604), The Mathworks Inc.).

An additional towed-camera transect was conducted over the complex topography of the hill (Fig. 4) using the SHRIMP vehicle^52^. Video footage was collected using a Bowtech Aquatech L3C-650 oblique-view, colour video camera mounted at the front of the vehicle. SHRIMP’s position was estimated from the ship’s position data, using a “layback calculation” based on the length of cable extending from the ship and the pressure reading from the vehicle. The resulting track was compared to the bathymetry collected by Autosub6000 to produce an error estimate^50^. The altitude of SHRIMP above the seabed was controlled by a winch operator using the live video feed as a reference.

### Image Selection and Processing

Photographs from the AUV surveys taken at an altitude of 1.9 m to 4.1 m above the seafloor were retained for analysis since this range provided the highest quality images. Images were processed using a custom Matlab script to correct for non-uniform illumination and to calculate the area surveyed based on the spatial positions of each photograph^29^. Since the oblique images overlapped by 80–85%, only every second image was included in the assessment. All vertical images within the given altitude range were analysed.

All selected images were visually inspected and any observed fish were identified to the most detailed taxonomic level possible based on their morphological characteristics^53^ using existing species lists for the region^21^,^53^. Individuals that could not be identified to species were identified to morphotype or recorded as indeterminate (indet. sp.). When the same individual was observed in sequential images, only the image in which it was closest to the AUV was included in the analysis.

Video footage from SHRIMP was examined and the identities of all observed fish were recorded to morphotype in the manner described above. The survey covered a total distance of c. 3,400 m, but the surveyed area could not be calculated due to variable topography and resulting uncertainty in the field of view. Fish counts from SHRIMP data are presented as counts (N) per linear km of survey.

### Bathymetric Data

High-resolution (5 × 5 m pixels) bathymetry data of the hill site were collected during research cruise RRS *Discovery* 377 using a Simrad EM2000 system mounted on the Autosub6000 AUV^50^. Lower-resolution (90 × 90 m pixels) broad-scale bathymetry of the wider PAP region was collected during RRS *James Cook* cruises 062^54^ and 071^55^ using the shipboard Simrad EM120 system. The CARIS Hips & Sips software was used for all bathymetric data processing^29^.

### Data Analyses

For all analyses, the significance level was set at α = 0.05. Fish densities were estimated by bootstrapping the fish counts and recalculating the density 10,000 times to produce estimates of the mean and 95% C.I.s using the package “boot”^55^ in R software^56^. All observed fish were retained in the dataset. Since different fish species may respond differently to the approaching AUV, differences in the fish community composition were compared between cameras using multivariate analyses in PRIMER 6 software^57^, following methods described in Clarke^58^. Analyses were based on both the untransformed density estimates of each species (individuals km^−2^) and presence-absence data. ANOSIM (Analysis Of SIMilarities) analyses based on Bray-Curtis similarity matrices were used to test for differences observed between the oblique and vertical cameras using 999 permutations. All photographs were randomly subdivided into four groups of equal size per camera to allow statistically-valid comparisons to be made. Where significant differences were detected, SIMPER (SIMilarity PERcentages) analysis was conducted to establish which taxa contributed most to the differences between cameras.

To determine whether distance from the abyssal hill had any effect on the distribution of the total fish fauna or of the dominant taxa, ArcGIS software (v. 10.1; http://www.arcgis.com) was used to map four depth contours onto the bathymetry of the abyssal hill at 4,800; 4,750; 4,700 and 4,650 m water depth (Figs 1 and 4). A series of raster grids (5 m resolution) were generated and used to calculate the shortest distance between every photograph and each of the four depth contours. The images were split into two groups, according to the presence or absence of fish. Fish counts from broad- and fine-scale surveys were combined for these analyses, but separated by camera type (Table 3). A bootstrapped Kolmogorov-Smirnov test (R package “Matching”^59^) was used to compare the distributions of the two groups. The significance level was estimated by bootstrapping the original count data 1,000 times and recalculating the Kolmogorov-Smirnov statistic to provide a measure of variance within the data.

To test the effects of elevated terrain on the densities of fish, the complete dataset was split into two groups containing images from “elevated” terrain (<4840 m depth) and images from the abyssal plain (>4840 m depth). Bootstrapped densities of the total fish fauna, macrourids, *Coryphaenoides* sp. 1 and *C. profundicolus* were calculated for each group. Differences were considered significant if the 95% C.I.s did not overlap.

Analyses of spatial distributions were conducted by using neighbour K statistics (broad-scale survey) and quadrat counts (fine-scale surveys). Given the large distances (1 km) between survey lines, a one-dimensional analysis^60^ was considered appropriate for the broad-scale surveys. Fundamentally, neighbour K statistics estimate the mean number of individuals within a given distance (*t*) along the AUV track of any other individual in the distribution. For the purposes of analysis, the survey lines were assumed to be contiguous. In the present study, the neighbour statistic was calculated at intervals of 100 images (c. 100 m for the vertical camera data and c. 200 m for the oblique camera data) using a custom R script. This interval was selected as a compromise between accuracy and the processing time required for significance testing. Significance testing was then conducted by comparing the observed data to a null distribution (representing a random distribution of individuals) generated by Monte-Carlo simulation, in which *N* images were randomly selected from the total dataset 1000 times for each distance interval *t* (where *N* is the number of individual fish observed). Observed statistical values that are greater than the null distribution indicate significant clustering at that distance, while those that are lower indicate significant dispersion at that distance. For ease of interpretation, the observed and simulated statistics have been normalised by the mean value for presentation in Fig. 3. This produces the standardised metric L(t), where a random spatial distribution is represented by L(t) = 0^60^. The distributions of the total fish counts and each of the two dominant taxa were assessed separately.

Each of the fine-scale surveys were treated as quadrats containing 11 × 11 cells, centred on the points at which the horizontal and vertical survey lines intersected. Each photograph was assigned to one of the 121 cells and the numbers of fish per cell were summed. These counts were used to calculate the observed variance to mean ratio of the fish in each quadrat (a common measure for estimating the spatial dispersion of individuals^37^). To test whether the observed ratio could have arisen by chance, Monte-Carlo simulations were used to randomly assign each photograph to a grid cell, recount the numbers of fish occurring per cell and estimate a distribution for the expected variance to mean ratio for each quadrat. This randomisation process was repeated 10,000 times per quadrat using a custom R script to generate 95% C.I.s.

## Additional Information

**How to cite this article**: Milligan, R. J. *et al*. High resolution study of the spatial distributions of abyssal fishes by autonomous underwater vehicle. *Sci. Rep.* **6**, 26095; doi: 10.1038/srep26095 (2016).

## Supplementary Material

Supplementary Information

## Figures and Tables

**Table 1:** Fish density (Autosub6000 surveys) and frequency (SHRIMP transect) estimates from seafloor images. Bootstrapped 95% C.I.s are given in parentheses for selected taxa.

Parameter	Density (individuals km^−2^)									Frequency (individuals km^−1^)
Survey	Broad Scale		North of hill (F1)	Hill flank (F2)		PAP benthic site (F3)		Total survey		Transect
Camera	Vertical	Oblique	Vertical	Vertical	Oblique	Vertical	Oblique	Vertical	Oblique	SHRIMP
All fish	801 (646,931)	409 (338,478)	767 (387,925)	679 (388,965)	539 (370,716)	662 (324,773)	382 (208,444)	763 (616,822)	423 (355,469)	3.24
All Macrouridae	748 (606,877)	368 (301,436)	738 (397,893)	614 (338,891)	512 (347,681)	638 (399,889)	360 (193,417)	712 (593,798)	388 (321,431)	2.65
Alepocephalidae sp. 1	–	–	–	–	–	–	11	–	2	–
*Bathysaurus mollis*	20	9	–	–	–	–	–	12	6	–
*Bassozetus* sp.	–	–	–	–	–	–	–	–	–	0.29
*Conocara* af. *salmoneum*	–	–	–	–	–	24	11	4	2	–
*Coryphaenoides armatus*	27	25	114	97	55	71	66	54	37	0.88
*Coryphaenoides profundicolus*	394 (288,488)	198 (149,248)	227 (52,347)	356 (145,564)	263 (150,394)	260 (106,419)	87 (20,132)	345 (266,408)	187 (147,223)	0.29
*Coryphaenoides* sp. 1	320 (231,411)	138 (97,179)	341 (108,460)	162 (19,303)	180 (85,287)	284 (126,448)	164 (64,218)	298 (234,357)	149 (112,179)	0.59
*Coryphaenoides* spp.	–	6	57	–	14	–	434	8	15	0.88
*Echinomacrurus* af. *mollis*	7	–	–	–	–	24	–	8	2	–
*Histiobranchus bathybius*	13	25	28	32	28	–	–	16	21	0.29
Zoarcidae sp.	7	–	–	32	–	–	–	8	–	–
Indet. sp. 1	–	3	–	–	–	–	–	–	2	–
Indet. spp.	13	3	–	–	–	24	–	12	2	–

**Table 2:** Fish density estimates from seafloor images taken on elevated topography (<4840 m depth) and on the abyssal plain (>4840 m depth). Bootstrapped 95% C.I.s are given in parentheses for selected taxa.

Parameter	Density (individuals km^−2^)			
Survey	Total survey: abyssal hill (4840–4768 m depth)		Total survey: abyssal plain (4852–4840 m depth)	
Camera	Vertical	Oblique	Vertical	Oblique
All fish	795 (583,1008)	411 (313,508)	723 (601,844)	354 (287,421)
All Macrouridae	765 (594,976)	393 (299,486)	660 (545,775)	319 (257,381)
Alepocephalidae sp. 1	0	0	0	3
*Bathysaurus mollis*	0	0	10	6
*Conocara* af. *salmoneum*	0	0	5	3
*Coryphaenoides armatus*	90	42	42	29
*Coryphaenoides profundicolus*	390 (239,540)	190 (125,256)	319 (239,399)	163 (118,208)
*Coryphaenoides* sp. 1	285 (157,413)	161 (101,221)	293 (216,369)	115 (77,152)
*Coryphaenoides* spp.	0	0	4	13
*Echinomacrurus* af. *mollis*	15	0	10	0
*Histiobranchus bathybius*	15	12	16	19
Zoarcidae sp.	15	0	16	0
Indet. sp. 1	0	6	0	0
Indet. spp.	0	0	16	9

**Table 3:** Summary data for the Autosub6000 surveys.

Survey No.	Survey scale	Depth Range (m)	Oblique Camera		Vertical Camera	
			No. Images	Area (km^2^)	No. Images	Area (km^2^)
B1	Broad	4806–4852	44,684	0.318	102072	0.150
F1	Fine	4847–4851	0	0	13,235	0.035
F2	Fine	4768–4820	10,488	0.072	47,967	0.031
F3	Fine	4846–4848	13,910	0.092	27,766	0.042
		TOTAL:	71,035	0.482	180,715	0.258
