# Supplementary material for: High resolution study of the spatial distributions of abyssal fishes by autonomous underwater vehicle
Source: Sci Rep. 2016 May 16;6:26095. doi: 10.1038/srep26095 (PMC4867640; doi:10.1038/srep26095)
Supplement: Supplementary Information [file srep26095-s1.pdf]

High resolution study of the spatial distributions of abyssal fishes by autonomous underwater vehicle - Supplementary Information

Authors and Affiliations: R.J. Milligan *, K.J. Morris, B.J. Bett, J.M. Durden, D.O.B. Jones, K. Robert, H.A. Ruhl, D.M. Bailey

Species Catalogue

Tables S1 and S2 contain exemplary images of the fish observed from the oblique-view and vertical-view cameras respectively. While the identities of certain taxa are likely to be correct (e.g. *Histiobranchus bathybius* and *Coryphaenoides armatus*), due to their distinctive morphology and the availability of good photographic guides, species identities should nonetheless be treated as tentative due to the difficulties in recognising certain taxonomic characteristics from photographs. All images have been cropped and had adjustments made to the contrast and colour levels for clarity using Photoshop CS5 software.

Comparisons to trawl-caught specimens suggest that *Coryphaenoides* sp. 1 was likely to be either *C. mediterraneus* or *C. leptolepis* based on body shape and colouration, but the species cannot currently be identified from photographs (Table S3).

Table S1: Exemplary oblique-view images of demersal fish and their identities.

**Image**	**I.D.**
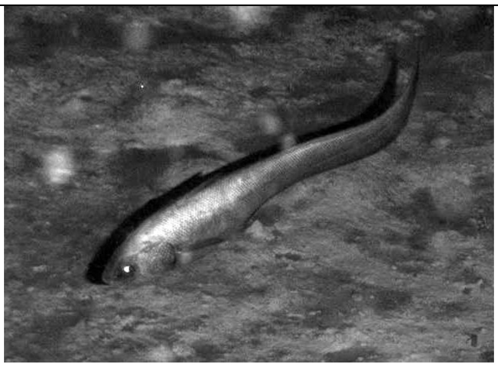 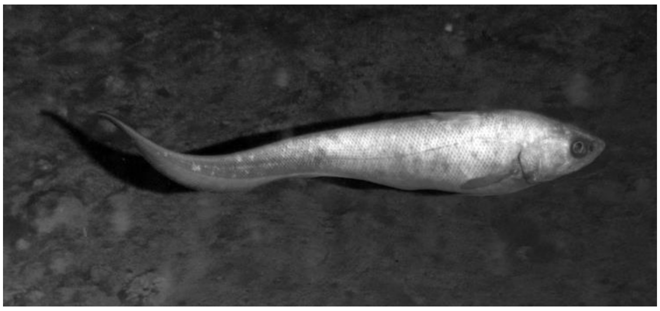	*Coryphaenoides armatus* (Hector, 1875)
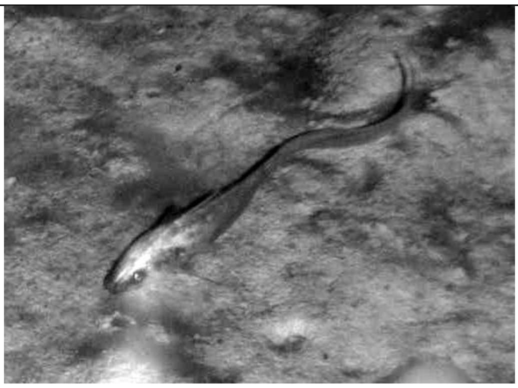 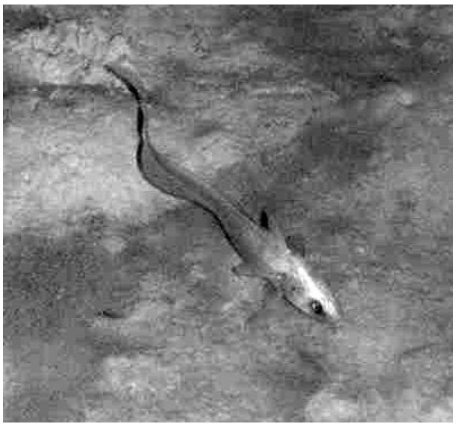	*Coryphaenoides* sp. 1

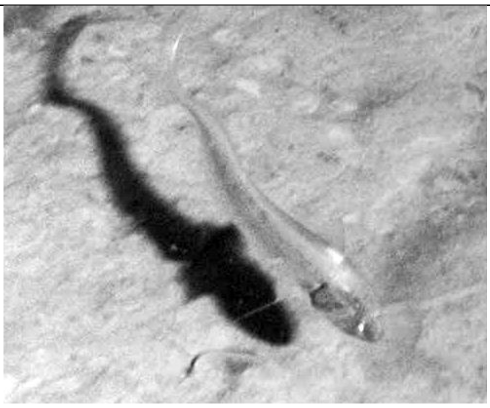 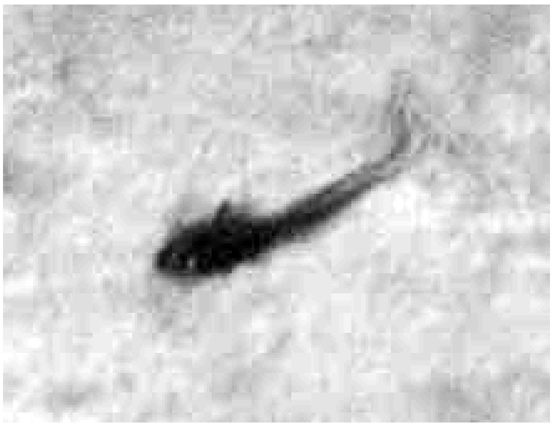	*Coryphaenoides profundicolus* (Nybelin, 1957)
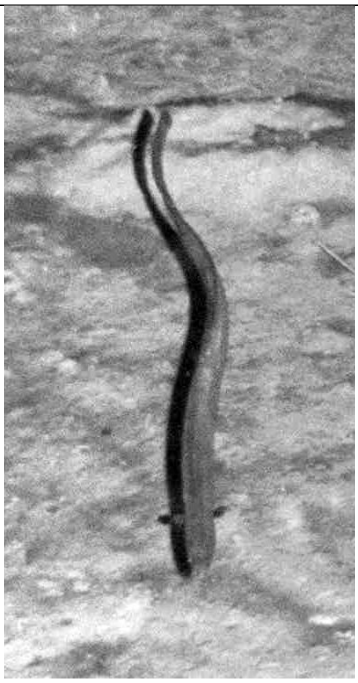	*Histiobranchus bathybius* (Günther, 1877)

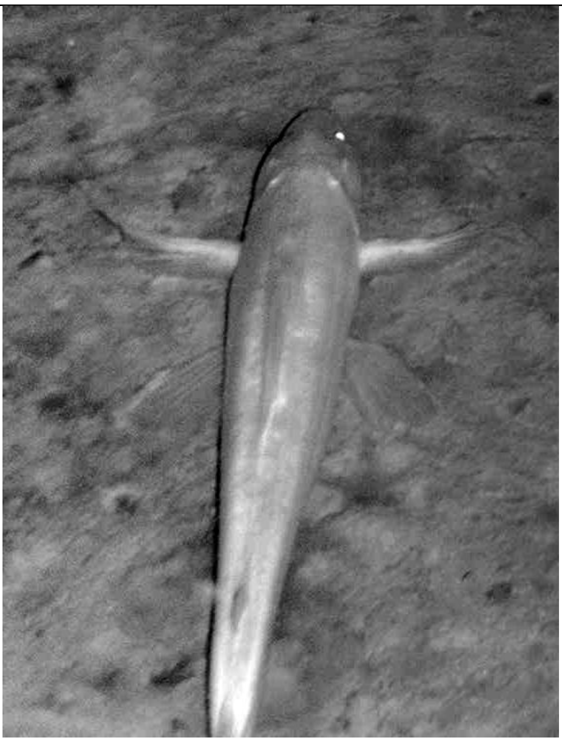	*Bathysaurus mollis* Günther, 1878
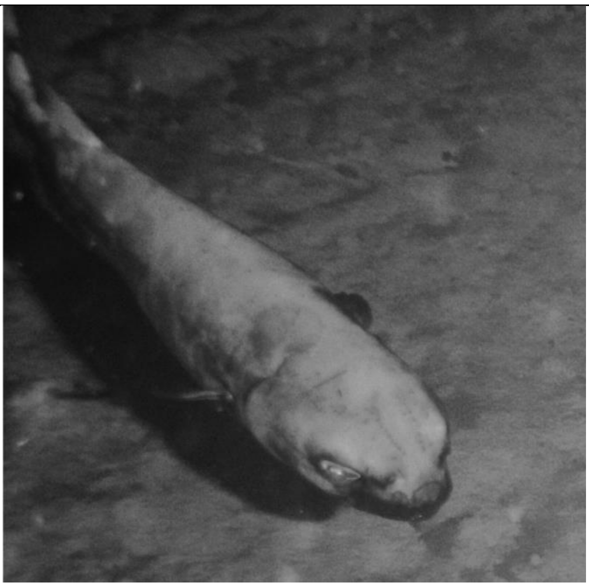	Alepocephalidae sp. 1

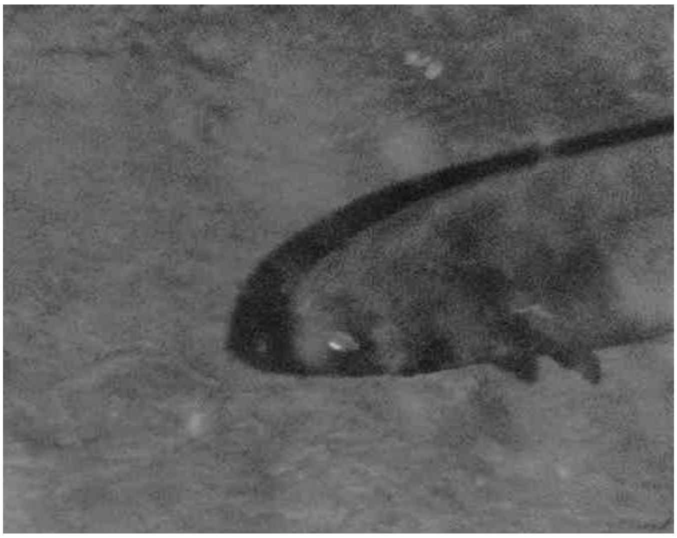	cf. *Conocara salmoneum* Gill & Townsend, 1897
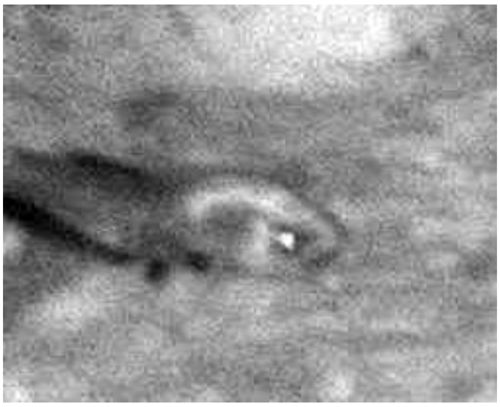	Indeterminate Sp. 1

Table S2: Exemplary vertical images of demersal fish and their identities.

Image	I.D.
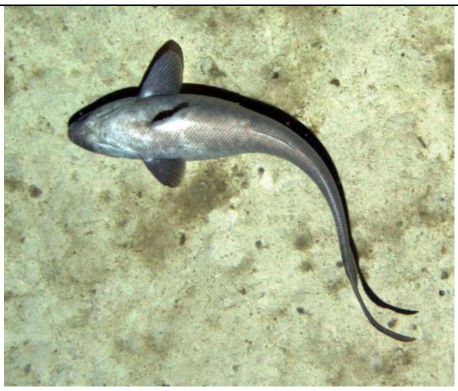	*Coryphaenoides armatus* (Hector, 1875)
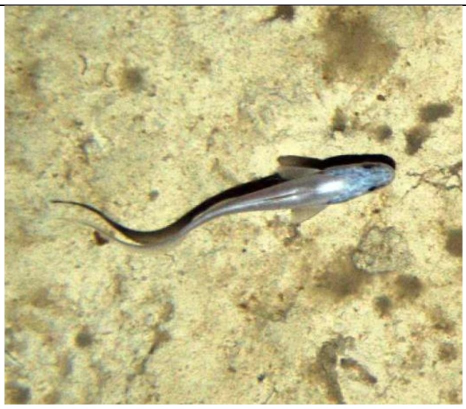 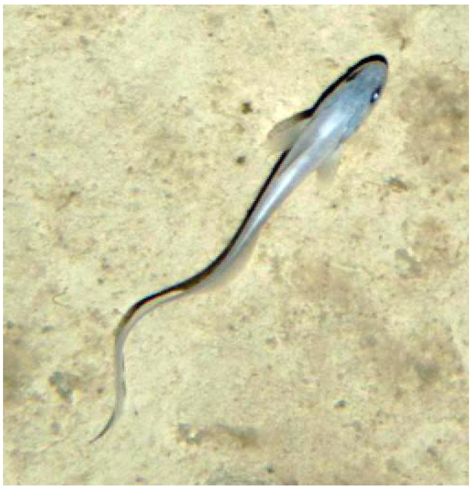	*Coryphaenoides* sp. 1

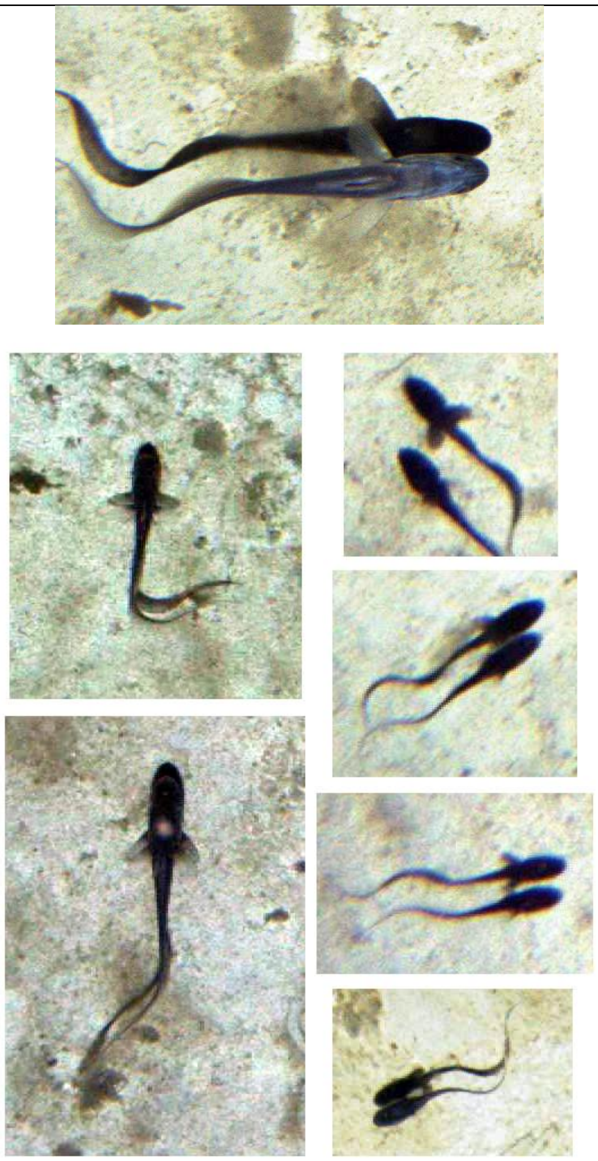	*Coryphaenoides profundicolus* (Nybelin, 1957)

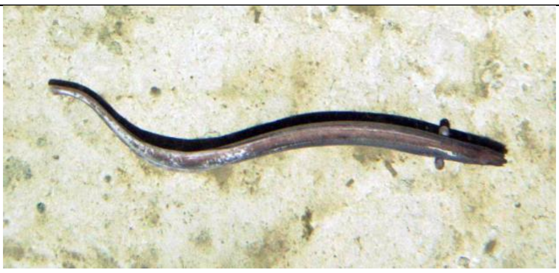	*Histiobranchus bathybius* (Günther, 1877)
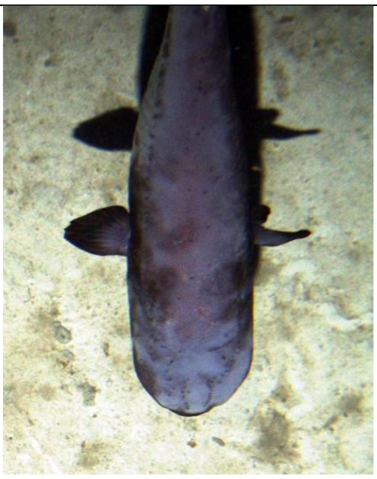	Cf. *Conocara salmoneum* Gill & Townsend, 1897
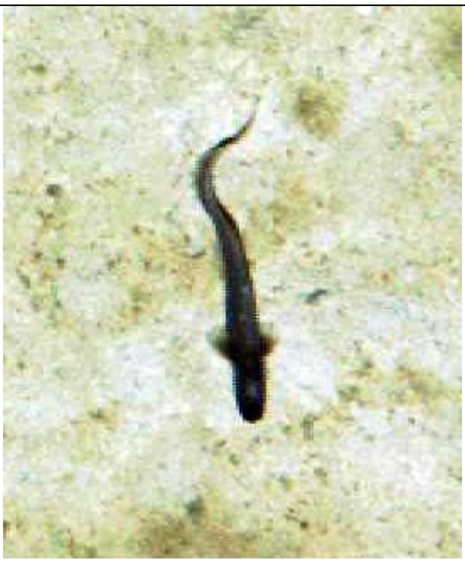	Zoarcidae

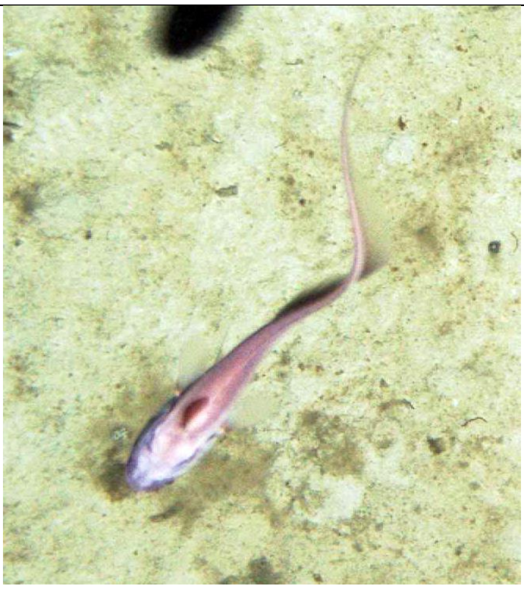	*Cf. Echinomacrurus mollis* Roule, 1916
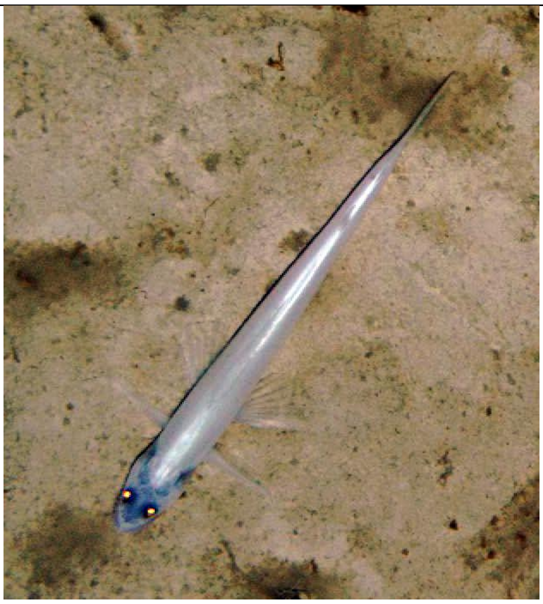	*Bathysaurus* sp.

Table S3: Example images of trawl-caught specimens of *Coryphaenoides mediterraneus, C. leptolepis* and *C. profundicolus* showing differences in colouration between species.

<table>
<tr><td>Image</td><td>I.D.</td></tr>
<tr><td>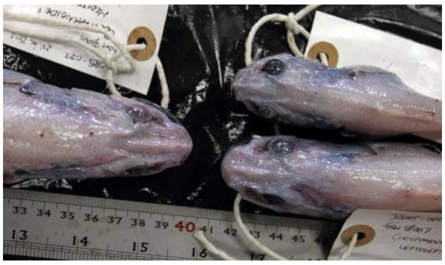</td><td>Comparison of C. mediterraneus (left) and C. leptolepis (right) dorsal head colouration.</td></tr>
<tr><td colspan="2">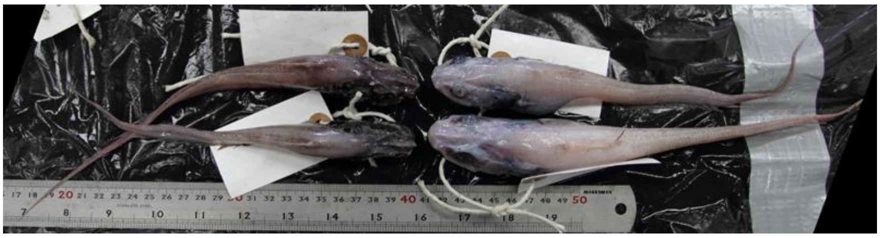
Comparison of C. profundicolus (left) with C. leptolepis (right) showing dorsal colouration.</td></tr>
</table>

Statistical Outputs

Kolmogorov-Smirnov Analyses

Tables S4-S6 show the results of the bootstrapped Kolmogorov-Smirnov analyses conducted for all fish, *Coryphaenoides* sp. 1 and *Coryphaenoides profundicolus* respectively. P-values were calculated from 1000 Monte Carlo simulations. In each case, the analyses tested for a distributional difference in the distance from the tested depth contours between those images containing fish and the ones that did not.

Table S4- Kolmogorov-Smirnov outputs (all fish)

Camera	Contour depth (m)	D statistic	p-value
Oblique	4800	0.046	0.82
	4750	0.050	0.75
	4700	0.049	0.77
	4650	0.030	0.99
Vertical	4800	0.120	0.83
	4750	0.153	0.55
	4700	0.118	0.85
	4650	0.124	0.80

Table S5 – Kolmogorov-Smirnov outputs (*Coryphaenoides* sp. 1)

Camera	Contour depth (m)	D statistic	p-value
Oblique	4800	0.060	0.98
	4750	0.080	0.81
	4700	0.074	0.88
	4650	0.062	0.97
Vertical	4800	0.242	0.60
	4750	0.272	0.45
	4700	0.301	0.32
	4650	0.311	0.29

Table S6 – Kolmogorov-Smirnov outputs (*Coryphaenoides profundicolus*)

Camera	Contour depth (m)	D statistic	p-value
Oblique	4800	0.127	0.12
	4750	0.110	0.24
	4700	0.122	0.15
	4650	0.106	0.28
Vertical	4800	0.252	0.34
	4750	0.286	0.20
	4700	0.251	0.34
	4650	0.236	0.42

Generalised Linear Models

Tables S7-S9 show the p-values generated by the binomial generalised linear models testing the effects of longitude, latitude and depth on the occurrence of all fish, *Coryphaenoides* sp. 1 and *C. profundicolus* respectively in the fine-scale surveys.

Table S7- Significance of each modelled variable on the occurrence of all fish

	**p-value**		
**Survey**	**Latitude**	**Longitude**	**Depth**
F2 (vertical)	0.59	0.96	0.38
F3 (oblique)	0.72	0.50	0.66
F3 (vertical)	0.09	0.79	0.98
F4 (oblique)	0.29	0.26	0.88
F4 (vertical)	0.72	0.92	0.33

Table S8- Significance of each variable on the occurrence of *Coryphaenoides* sp. 1

	**p-value**		
**Survey**	**Latitude**	**Survey**	**Latitude**
F2 (vertical)	0.56	0.26	0.52
F3 (oblique)	0.49	0.98	0.62
F3 (vertical)	0.18	0.83	0.95
F4 (oblique)	0.25	0.49	0.74
F4 (vertical)	0.53	0.18	0.43

Table S9- Significance of each variable on the occurrence of *Coryphaenoides profundicolus*

	**p-value**		
**Survey**	**Latitude**	**Survey**	**Latitude**
F2 (vertical)	0.24	0.11	0.07
F3 (oblique)	0.56	0.11	0.45
F3 (vertical)	0.62	0.76	0.98
F4 (oblique)	0.81	0.37	0.82
F4 (vertical)	0.26	0.67	0.75
